# Reactions of Surface-Confined Terminal Alkynes Mediated by Diverse Regulation Strategies

**DOI:** 10.3390/nano15161271

**Published:** 2025-08-18

**Authors:** Yun Wu, Lei Xu, Junxi Li, Chi Zhang

**Affiliations:** Interdisciplinary Materials Research Center, School of Materials Science and Engineering, Tongji University, Shanghai 201804, China

**Keywords:** terminal alkyne, on-surface synthesis, low-dimensional nanostructure, scanning probe microscopy

## Abstract

Terminal alkynes, characterized by *sp*-hybridized carbon atoms at the molecular termini, possess high electron density and exceptional chemical reactivity. These properties make them ideal candidates for the synthesis of one-dimensional molecular wires and two-dimensional networks. Advances in nanoscale characterization techniques, such as scanning tunneling microscopy and atomic force microscopy, have enabled the real-space visualization of molecular assembly and chemical reactions of terminal alkynes and in situ atomic-level manipulations under surface-confined conditions. In addition, through the combination of spectroscopic measurements, physicochemical properties of and information about resulting nanostructures have been achieved. Moreover, density functional theory calculations provide deeper insights into the underlying reaction pathways and mechanisms. From this perspective, this review summarizes recent progress in the assembly and chemical transformations of terminal alkynes on noble metal surfaces. It discusses strategies for structural modulation and reaction selectivity control, including direct incorporation of heteroatoms or functional groups into precursors, the selection of metal surfaces, the introduction of extrinsic components into molecular systems, and atomic-scale manipulations using scanning probes.

## 1. Introduction

Carbon, the elemental backbone of organic matter, serves as a cornerstone of organic chemistry and materials science due to its unparalleled ability to form versatile *sp*/*sp*^2^/*sp*^3^ hybridized architectures [1,2,3]. This unique attribute enables the creation of structurally diverse allotropes and compounds, driving revolutionary advancements in synthetic chemistry, sustainable chemistry, nanoelectronics, etc. In particular, the synthesis of carbon nanomaterials with precisely designed functionalities has emerged as a critical frontier, directly addressing global challenges in energy storage [4,5,6], quantum computing [7,8], and environmental remediation [9,10,11]. Among these, graphdiyne has appeared as a promising carbon-based nanomaterial, featured by its “–C≡C–C≡C–” linkages [12,13]. Theoretical studies indicate that it possesses high carrier mobility and a tunable bandgap [12,14], positioning it to bridge the zero-bandgap graphene and conventional semiconductors. However, due to the inherently high reactivity of alkynyl groups, the synthesis often deviates from the predetermined pathways. Consequently, the fabrication of large-scale and high-quality graphdiyne structures presently faces persistent challenges, necessitating optimal molecular precursors and reaction conditions for its controlled synthesis.

Terminal alkynes (TAs, R–C≡C–H), distinguished by their exposed *sp*-hybridized carbon termini while being different from internal alkynes (R_1_–C≡C–R_2_) constrained by adjacent groups and electronic delocalization, are considered as precursors for the synthesis of diynes [15,16] or even graphdiynes [17] and powerful components to build manifold predefined structures for nanocarbon engineering [18]. At the same time, the high reactivity of terminal alkynes also leads to the formation of byproducts during reactions [19]. In order to further investigate the reaction pathways during synthesis and elucidate the reaction mechanisms to control reaction selectivity, characterization techniques with atomic-level precision serve as power tools.

In 1982, Binnig and Rohrer obtained the surface topography by scanning tunneling microscopy (STM) [20]. Since then, this disruptive creation has provided a paradigm shift in the atomic-level understanding of nanomaterials and opened up a new era of surface science. Along with the rapid development of STM [21], another invention, atomic force microscopy (AFM) [22] came into being, which is capable of characterizing almost any type of surface, while STM is only applicable to conductors and semiconductors. Nowadays, by means of tip functionalization and the combination of various nanosensors [23,24], like qPlus force sensors and CO-modified tips, a scanning probe microscope (SPM) family with numerous variations has been constructed systematically. It thus allows researchers to realize atomic-resolution imaging and bond-order analysis [23,25], image the molecular adsorption configurations and reaction process on surfaces [26,27], unravel the physicochemical properties of surface nanostructures [28,29], manipulate molecular nanostructures and trigger reactions in situ [29,30,31,32,33,34], etc. Thanks to the continuous progress in SPM, a sub-divided field termed on-surface synthesis has emerged, aiming at constructing predefined low-dimensional nanostructures based on solid surfaces as confinement templates and catalysts [35,36,37,38].

Compared to conventional solution-phase synthesis, on-surface synthesis depends on the aforementioned SPM techniques complemented by spectroscopic measurements such as X-ray photoelectron spectroscopy (XPS) and density functional theory (DFT) calculations. It involves precursor design to form reactant assembly, intermediate state capture, and reaction products’ characterization, which are realized exquisitely through a bottom-up way. Simultaneously, on-surface synthesis provides the possibility to overcome the difficulties in the poor solubility of reactants in solution synthesis. Moreover, the catalytic effect of metal substrates facilitates on-surface reactions under mild conditions, which would otherwise have high reaction barriers in solution. Furthermore, the confinement and template effects of substrates can improve the reaction selectivity, stabilize some reactive intermediates (e.g., surface-stabilized radicals [39,40]), and may also induce different reaction pathways [41,42,43].

In addition, on-surface synthesis depends crucially on the design of molecular precursors and varieties of interactions involved in the molecule-surface systems. In other words, size, topology, mobility, chirality, symmetry, functional groups, and other such attributes are essential design parameters [44,45,46]. In 2007, Grill and his colleagues realized Ullmann-type coupling reactions via thermal activation and modified the numbers and sites of bromines functionalized in molecular precursors to controllably construct covalent zero-dimensional dimers, one-dimensional chains, and two-dimensional networks [47]. This pioneering work demonstrates that thermal treatment can effectively induce on-surface reactions based on rational precursor design.

In order to reduce reaction barriers and optimize reaction pathways to construct novel and stable product frameworks, diverse regulatory strategies need to be adopted, such as on-surface reactions steered by pre-assembly [48,49,50]. Molecular assembly is dictated by non-covalent intermolecular interactions such as hydrogen bonding [51,52], coordination bonds [53], electrostatic interactions [54], π-π stacking, etc. Despite their individually weak bonding nature, non-covalent interactions exhibit more extended effective ranges at the atomic scale compared to covalent bonds. Through collective interplay, these interactions can direct reaction selectivity by steering molecular orientations and transition-state stabilization in a surface-confined environment. Nevertheless, such intrinsic interactions are insufficient for precise synthesis. Recent studies have demonstrated that extrinsic components can serve as potent modulators [55] by activating inert chemical bonds [56,57], directing dynamic equilibrium of covalent products [58,59], and isolating or removing byproducts [60,61]. Recently, a radical transfer mechanism [40,62] once again displayed that extrinsic components can effectively regulate the reaction pathways of terminal alkynes by facilitating C–H activation of terminal alkynes. This molecular-level programmability addresses the longstanding trade-off between structural integrity and functionality in nanocarbon systems.

**Scheme 1:**
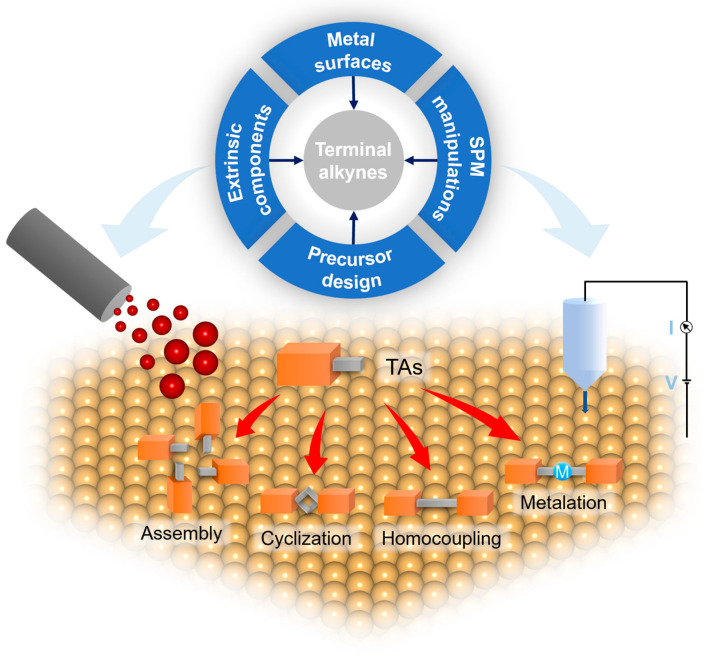
Illustration of the assembly and reactions of terminal alkynes on surfaces mediated by diverse regulation strategies.

In this review, we summarize on-surface assembly and reactions of terminal alkynes. This review classifies diverse regulatory strategies, including supramolecular assembly, selection of metal substrates, precursor design, tip manipulation, and extrinsic component introduction in individual sections (Scheme 1). Based on the influence of different strategies on the reactivity and selectivity of terminal alkynes, we also discuss the key to such successful regulation and elucidate underlying reaction mechanisms. Finally, we briefly summarize the guiding role of these regulatory strategies and provide an outlook on the current challenges and future research directions. The exciting progress shown in this review highlights the transformative potential of surface-confined terminal alkynes in synthesizing carbon-based nanomaterials, transcending the limitations of conventional solution-phase synthesis.

## 2. Supramolecular Assembly of Terminal Alkynes

**Figure 1:**
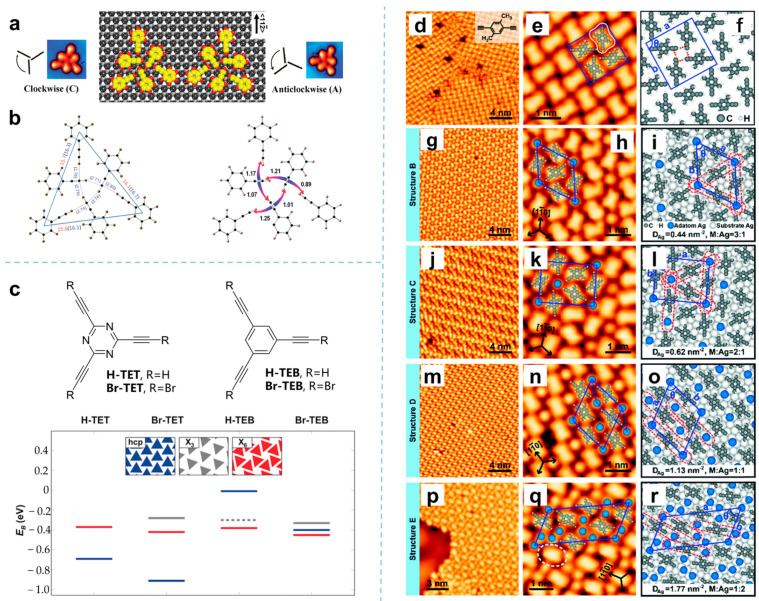
Supramolecular self-assembly of terminal alkynes on surfaces. (**a**) Clockwise (C) and anticlockwise (A) chiral adsorption configuration of hexamers aggregated by six phenylacetylene molecules through C–H∙∙∙π interactions. (**b**) DFT-calculated structural model and bonding analysis of NBO. Arrow color indicates electron donor (blue) or electron acceptor (red) behavior. Copyright 2012 American Chemical Society [63]. (**c**) Binding energy of different triethynyltriazine derivatives. Blue, grey, and red solid lines represent hcp, X_3_-synthon, and X_6_-synthon, respectively. Grey dashed lines indicates dense X_3_-synthon. Copyright 2020 The Authors. Published by Wiley-VCH Verlag GmbH & Co., KGaA [51]. (**d**–**r**) STM images along with their magnified views and structural models corresponding to low-temperature deposition, RT annealing for 15, 40, 60 min, and RT deposition, respectively. Structural motifs are highlighted by red dashed circles, and blue parallelograms denote the unit cells of Ag adatoms. Copyright 2016 The Royal Society of Chemistry [64].

Supramolecular self-assembly is a process where two or more randomly distributed molecules integrate together via intermolecular non-covalent interactions, resulting in well-organized nanostructures without external disturbance [48]. Despite the dominance of covalent bonds in chemical structures, supramolecular interactions are non-negligible regulators for molecular aggregation, especially under non-equilibrium conditions. This implies that assembled structures of molecular precursors which are mainly dependent on functional groups would play an essential role in reaction processes. As for terminal alkynes, the carbon–carbon triple bond serves as a dual-functional moiety: its electron-rich π-system enables directional C–H∙∙∙π contacts with electron-deficient regions of adjacent molecules, while its linear structure further facilitated terminal H to interact with the electron-rich triple bond. For instance, ethynyl group functionalized benzene (i.e., phenylacetylene) was chosen by Li et al. [63] to introduce attractive interactions to construct supramolecular clusters with chirality on Au(111) (Figure 1a). Such clusters were dominated by directional C–H∙∙∙π bonding instead of intermolecular repulsion resulted from a 2D-confined π-conjugated system arranged in a relative parallel way. Within the chiral triangular units, three centrally positioned molecules adopt a pinwheel conformation mediated by cooperative C–H∙∙∙π interactions, with ethynyl groups acting as both electron acceptors and donors. Such an interaction scenario was theoretically confirmed by DFT calculations and Natural Bond Orbital (NBO) analysis (Figure 1b).

In addition to the C–H∙∙∙π interaction, hydrogen and halogen bonding contributed by H-terminated and Br-substituted terminal alkynyl groups are also pivotal for fabricating large supramolecular networks. Maier and co-workers [51] selected triethynyltriazine (TET) and triethynylbenzene (TEB) derivatives as shown in the upper panel of Figure 1c to prepare graphyne-like supramolecular networks on Ag(111) and Au(111) via N∙∙∙H–C(*sp*) and N∙∙∙Br–C(*sp*) bonds formed between alkynyl groups and triazine cores. According to the calculated binding energies shown in the lower panel of Figure 1c, they found that TET-based monomers exhibited preferential stabilization in hcp configurations, whereas TEB-based counterparts had thermodynamic preference for X_6_-synthon structures. In addition, structures composed of Br–TET/TEB are more stable than those composed of H–TET/TEB, indicating the role of N-modification and Br-terminated alkynyl groups in the construction of supramolecular networks. Moreover, the interaction between the terminal alkyne 1,4-diethynyl-2,5-dimethylbenzene (DEDM) (as shown in the inset of Figure 1d) and metal adatoms was embodied in Ag-alkyne co-assembled structures as prepared by Wu’s group [64]. In this work, tunable Ag adatom density was achieved by strategically employing thermodynamic parameter modulation (i.e., annealing duration and deposition temperatures), leading to the construction of various assembled nanostructures as shown in Figure 1d–r. DEDM molecules, which were deposited at low temperature and annealed at room temperature (RT), formed a series of co-assembled structures with increasing Ag adatom density through prolonged annealing time. Moreover, when deposited at RT directly, DEDM yielded a higher Ag adatom density co-assembled structure. The DFT-calculated charge-density differences further elucidated the substrate-mediated charge localization between Ag adatoms and the terminal alkynyl groups stabilizing the DEDM–Ag(111) system. Such a study thus highlights the role of surface adatoms in the modification of nanostructures of terminal alkynes.

Inspired by the introduction of adjunctive functional groups and thermodynamic control as mentioned above, pre-assembly structures of terminal alkynes on metal surfaces can be further orchestrated to steer their on-surface reactions through three synergistic operational modalities: regulating reaction selectivity, steering reaction pathways, and spatially constraining active sites [49,65,66,67]. For instance, Li et al. reported low-temperature selective synthesis of tetraphenyl[4]radialene through the tetramerization of phenylacetylene molecules on a Cu(100) surface [67]. Mechanistic investigation reveals a concerted [1+1+1+1] cycloaddition pathway mediated by a quadruple C–H∙∙∙π-directed assembly of phenylacetylene, providing a feasible method to synthesis [4]radialenes.

Therefore, predesigned supramolecular assembly constitutes a viable synthetic strategy, especially in the cases where terminal alkynyl moieties exhibit a pronounced capability for directional non-covalent bonding. This assembly mainly arises from cooperative C–H∙∙∙π interactions and hydrogen bonds, which dominate their supramolecular association behavior.

## 3. Reactions Regulated by Metal Surfaces

The supporting metal substrates generally influence on-surfaces synthesis, where the reactivity and reaction selectivity of molecular precursors are principally governed by the chemical nature and lattice parameters of the underlying substrates [36,68]. This mechanistic framework enables divergent reaction products from identical precursors through substrate selection [69,70], establishing a feasible platform for steering on-surface reactions. The regulatory efficacy hinges on precise optimization of interfacial binding energy while maintaining molecular surface mobility—a balance critically influenced by substrate coordination geometry. Inert surfaces exhibiting weak interfacial coupling usually promote adsorbate surface diffusion, with crystallographic plane-dependent surface inertness following the hierarchy: (111) > (hkl) > (110), inversely correlating with catalytic activity metrics [36].

**Figure 2:**
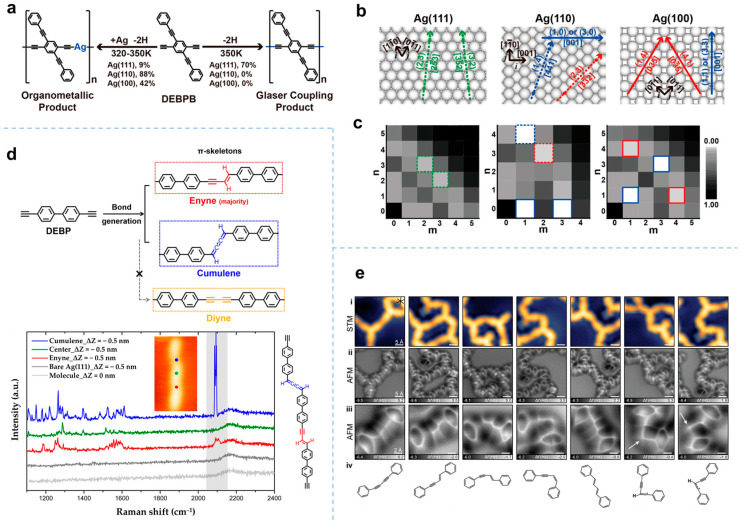
Reactions of terminal alkynes on different Ag surfaces. (**a**) DEBPB reaction pathway regulated by Ag(111), Ag(110), and Ag(100). (**b**,**c**) Quantifying lattice commensurability between organometallic chain along different directions and substrate lattices. The colored arrows mark directions where the lattice constants show a relatively good match with the organometallic chain’s periodicity. The brighter the (m,n) square, the better the degree of lattice matches. Copyright 2015 American Chemical Society [71]. (**d**) Chemical bond formation and identification from DEBP on Ag(111). Copyright 2021 American Chemical Society [72]. (**e**) Identification of post-400K-annealed products of Ext-TEB on Ag(111). (**i**–**iv**) STM images, AFM images, enlarged AFM images, and chemical structures. The white arrows indicate a particular structure at approximately 90 degrees. Copyright 2024 The Authors. Advanced Materials Interfaces published by Wiley-VCH GmbH [73].

As an illustrative example, the facet-dependent reaction selectivity of 2,5-diethynyl-1,4-bis(phenylethynyl)-benzene systematically investigated on Ag(111), Ag(110), and Ag(100) substrates (Figure 2a–c) [71] was chosen herein. Ag(111) surfaces mainly drove the homocoupling of molecules, yielding one-dimensional covalent molecular wires, while organometallic (OM) molecular wires were dominated on Ag(110) and Ag(100) facets instead. The pronounced product selectivity (88% for OM product) observed on Ag(110) arose from the perfect match between the periodicity of OM molecular wires and the crystallographic parameters of the substrate.

Due to the high reactivity of terminal alkynes, enormous efforts have been mainly devoted to exploring the reactions of terminal alkynes [74,75] and further identifying their products on Ag(111). Unambiguously, by a combination of STM/STS and tip-enhanced Raman spectroscopy (TERS) techniques, Kim et al. chemically identified and controlled the generated π-skeletons during alkyne coupling processes [72]. Specifically, the 4,4′-diethynyl-1,1′-biphenyl (DEBP) molecule exhibited preferential formation of enyne moieties by thermal treatment, with significantly suppressed cumulene and the complete absence of diyne moieties (Figure 2d). This thus confirmed the non-dehydrogenative C–C coupling scenario instead of the dehydrogenative Glaser one, which is also in good agreement with the work by de Oteyza on Au(111) [76]. Moreover, high-resolution bond-resolved nc-AFM was employed in a later work to recognize and characterize the coupling reaction product structures of terminal alkynes on Ag(111) (Figure 2e). It then unraveled that 1,3,5-tris-(4-ethynylphenyl)benzene (Ext-TEB) molecules predominantly yielded enyne bonding at 350 K with minor byproducts (e.g., butadiyne and butadiene derivatives), while rare dehydrogenative coupling was formed after further annealing to 400 K [73]. These efforts eventually reassessed the reaction products, scenarios, and corresponding mechanisms of alkyne coupling on some typical substrates such as Ag(111) and Au(111).

Despite the prevalence of side reactions of terminal alkynes under thermal activation on Ag(111), the utilization of Ag(877) surfaces [77] markedly enhanced the reaction selectivity by aligning molecular orientations at step edges. This alignment enabled the controlled synthesis of extended π-conjugated nanowires with lengths exceeding 30 nm. Additionally, de Oteyza and his co-workers demonstrated that Ag(100) surfaces can facilitate Bergman cyclization of rationally designed alkynes (i.e., containing enediyne moiety) [78]. These findings collectively establish the critical role of surface lattices in dictating the selectivity of on-surface synthesis.

**Figure 3:**
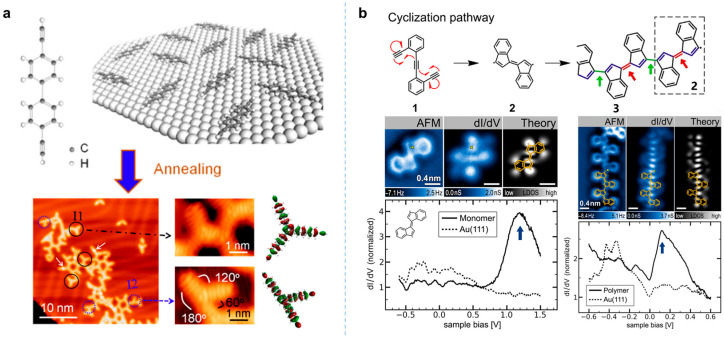
Reactions of terminal alkynes on Au(111). (**a**) Cyclotrimerization of diyne monomer. The black solid circles and blue dashed circles respectively represent two connection modes. Copyright 2014 American Chemical Society [80]. (**b**) Thermal activation reaction pathway of 1,2-bis(2-ethynylphenyl)ethyne and electronic structure of its isolated building block 2 and an individual oligomer chain. The blue arrows point out the monomer LUMO and an electronic resonance, respectively. Copyright 2014 American Chemical Society [81].

Elemental selection of metallic substrates constitutes a critical regulation strategy for surface-mediated synthesis. The low reactivity of gold surfaces hinders the formation of organogold structures and favors the cyclization of terminal alkynes into a stable cyclic compound [79]. On Au(111), diyne monomers (DEBP) [80] underwent [2+2+2] cyclotrimerization to form two-dimensional covalent networks with predominant 1,3,5-cyclization products and minor 1,2,4-cyclization ones due to steric hindrance (Figure 3a). Interestingly, for a structurally distinct enediyne precursor [81], the Bergman cyclization reaction took place on Au(111), enabling the synthesis of one-dimensional oligo-(E)-1,1′-bi(indenylidene) chains with extended π-electron delocalization and periodic bond-length alternation (Figure 3b). Furthermore, the template effect of Au(110) suppressed 1,3,5-trimerization while favoring 1,2,4-trimerization and homocoupling [82].

Given the different catalytic reaction performances of metal surfaces, various types of metal substrates were selected and compared in several previous works [36,37,83]. For example, Ji et al. conducted a control experiment analysis on the reaction pathways of 5,10,15,20-tetra(4-ethynylphenyl)porphyrin on Au(111) and Ag(111) [84], revealing that cyclodimerization predominated on Au(111), while enynes were the main products on Ag(111). As for Cu substrates, due to their inherently higher reactivity, the formation of organometallic complexes with adsorbed molecular species could be promoted [85].

Therefore, these studies demonstrate that surface-confined reactions of terminal alkynes are fundamentally governed by substrate parameters, including the crystallographic plane, elemental composition, and interfacial property. This also establishes substrate engineering as a feasible strategy for steering on-surface molecular reactions.

## 4. Reactions Regulated by the Precursor Design

As on-surface synthesis crucially relies on well-designed molecules serving as building blocks, which further connect with each other and construct molecular nanostructures from bottom to top, rational design of molecular precursors is essential to control the reaction pathways and products. Accordingly, additional functional groups have been incorporated into terminal alkynes [86,87,88,89,90], and heteroatoms have been inserted to aromatic skeletons instead of carbon atoms [51,91]. As a result, the modulation of reaction selectivity, lowered activation energy, and stepwise reaction control [92,93] can be achieved.

**Figure 4:**
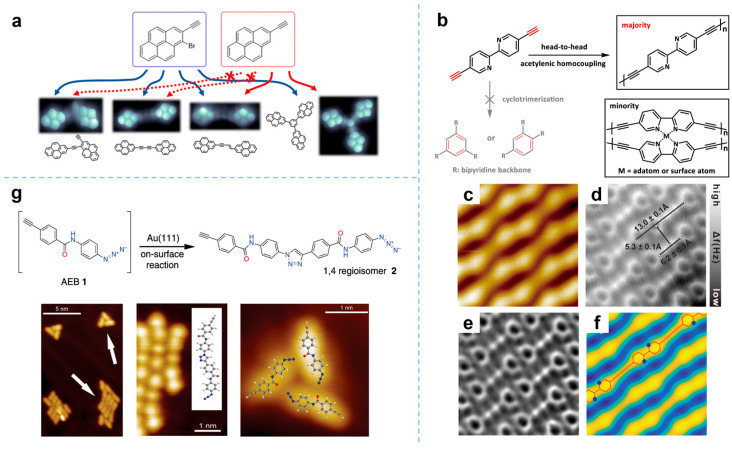
Reactions of terminal alkynes regulated by precursor design. (**a**) Different reaction pathways of halogen-functionalized versus unfunctionalized terminal alkynes on Au(111). Copyright 2021 American Chemical Society [76]. (**b**–**f**) Highly selective formation of graphdiyne chains from nitrogen-doped terminal alkynes on Au(111). Copyright 2023 American Chemical Society [91]. (**g**) Reaction scheme of AEB 1 and STM characterization of 1,4-regioisomer 2 and persistent ternary non-covalent monomer assembly. Copyright 2013 American Chemical Society [90].

A typical example is the coexistence of terminal alkynyl groups and halogen functional groups within a single molecule, allowing diverse reaction pathways, including Ullmann coupling, Sonogashira coupling, and Glaser-type coupling. Interestingly, the 1-bromo-2-ethynylpyrene precursor was selected by de Oteyza’s team [76] to study the selectivity of a reaction pathway upon annealing on Au(111). As shown in Figure 4a, this precursor was demonstrated to undergo Glaser coupling, yielding diyne structures along with other side products such as enyne structures, fused trimeric structures with subsequent temperature-dependent cyclization, and single alkyne structures (via Sonogashira reaction). Notably, control experiments using non-brominated precursors unequivocally established that C–Br groups were indispensable for the formation of the diyne structure. In their absence, alkyne coupling exhibited high selectivity toward non-dehydrogenative head-to-head addition to form an enyne without a diyne product, which is in line with the reaction scenario on Ag(111) as discussed above [72].

Moreover, the introduction of halogen functional groups into terminal alkyne molecules reduces the reaction barrier to some extent. Comparative deposition of iodine-free ethynyl-phenanthrene (EP) and iodinated ethynyl-iodophenanthrene (EIP) onto Au(111) revealed that EP remained intact, whereas EIP underwent partial deiodination [87]. This process generated radicals that promoted alkyne activation, ultimately yielding linear polymer chains extending up to ~15 nm. Wu et al. [93] achieved a stepwise dissymmetric reaction by introducing dual bromine substituents on the molecule featuring bis-terminal alkynes. Annealing treatment at 300 K induced the removal of the first bromine, generating alkynyl–silver–alkynyl linkages that form linear chains. Concurrently, residual bromines mediated interchain halogen bonding, yielding an ordered arrangement. Subsequent annealing within the range of 320–450 K facilitated dissociation of the second bromine, enabling the formation of additional alkynyl–silver–phenyl connections. These combined interactions cooperatively constructed a highly ordered two-dimensional organometallic network.

Another strategy, pyridinic nitrogen modification in place of carbon atoms, was proposed by Chi’s group to produce linear nanowires on Au(111) with high selectivity [91]. The 5,5′-diethynyl-2,2′-bipyridine was chosen as the molecular precursor to suppress both 1,3,5- and 1,2,4-ring trimerization pathways and promote highly selective homocoupling reactions of terminal alkynes. In such a way, well-aligned N-doped π-conjugated nanowires were prepared, as characterized by both STM and nc-AFM (Figure 4b–f). In addition to the aforementioned Sonogashira reaction (i.e., the coupling reaction of terminal alkynes with aryl halides), the azide–alkyne cycloaddition, as the most common “click” reaction, has also aroused researchers’ attention. Fuchs’s group studied this reaction on Au(111) [90] by applying N-(4-azidophenyl)-4-ethynyl-benzamide (AEB 1) (see Figure 4g) as the molecular precursor, in which two benzene rings linked by an amide act as the backbone and azide and alkyne are at two opposite ends of it to induce a cycloaddition reaction. The amide linker promoted planar adsorption and enhanced the surface diffusion of monomers, which is beneficial for the two functional groups to further meet and react with each other. The amide linkage also facilitated intermolecular hydrogen bonding orthogonal to the reaction axis, thereby resulting in enhanced structural ordering of the post-reaction molecular assembly and improved reaction possibility. Compared to the 1,3-diploar cycloaddition, the 1,4-regioisomer 2 was prepared successfully, while 1,5-regioisomer 3 was absent due to the two-dimensional confinement of Au(111).

Therefore, rational precursor design, through strategic incorporation of functional groups or heteroatoms, demonstrates their capacity in regulating reaction selectivity, kinetics, and ultimate nanostructures.

## 5. Reactions Regulated by Tip Manipulation

The advancement of SPM not only facilitates surface characterization but also enables atomic- and molecular-scale manipulation at designated sites through bias voltages or mechanical force application to the probe tips [30,32,94,95,96]. At the nanoscale, vibrational modes of target atoms or molecules can be excited, when the tip approaches them [24], inducing subsequent diffusion [31,33,34], desorption [29], selective bond dissociation and formation [26,97,98], etc. In general, tip manipulation can be divided into two modes. One is vertical manipulation [99,100], where atoms or molecules adsorbed on the substrates are lifted by a tip, moved to a target position, and then released by breaking the tip-atom/molecule to complete the manipulation. The other is lateral manipulation [34], in which the tip mechanistically disturbs atoms or molecules strongly enough to overcome the diffusion barriers, enabling direct pushing or pulling to move them across the surfaces.

**Figure 5:**
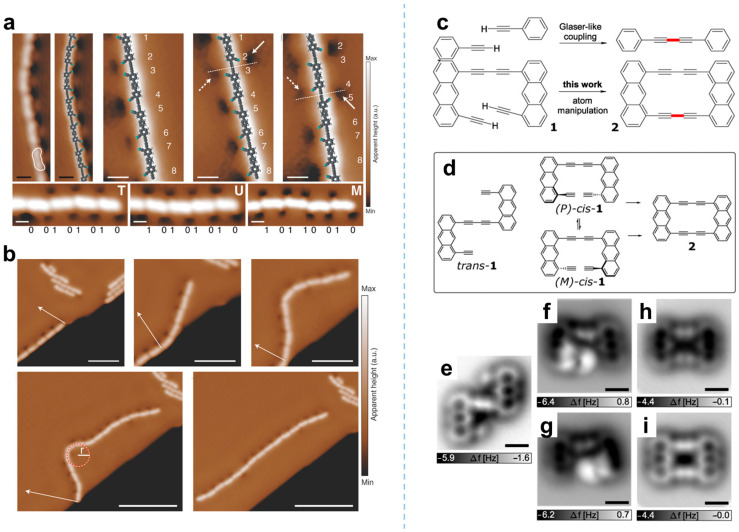
Reactions of terminal alkynes regulated by tip manipulation. (**a**) Conformational reversal induced by tip manipulation. The lower panel represents “T”, “U”, “M” encoded 8-bit ASCII values, respectively. Scale bars are 10 Å. (**b**) Flexibility of nanowires characterized through tip manipulation. The scale bars in upper and lower panels are 50 Å and 100 Å, respectively. The white arrows indicate the displacement of the tip during manipulation. Copyright 2018 WILEY-VCH Verlag GmbH & Co. KGaA, Weiheim [101]. (**c**) Schematic illustration of tip-induced Glaser coupling. (**d**) Conformations of precursor 1 and product 2. (**e**–**g**) AFM images of precursor 1 in different conformations. Scale bars are 5 Å. (**h**,**i**) AFM images of product 2 obtained at different tip heights. Scale bars are 5 Å. Copyright 2020 The Authors. Published by Wiley-VCH GmbH [102].

A nice example is shown in Figure 5a,b. Based on the terminal alkyne precursors functionalized by double cyano (CN) groups, 40 nm long π-conjugated nanowires were synthesized on a Ag(455) surface template [101], with each CN group encoding a single bit of information (Figure 5a). The stored information in a single nanowire can be further modified by tip manipulation. The tip was first aligned with the black dent caused by the target CN group (indicated by the white arrow), and then it approached the surface until it connected to the CN group confirmed by a sudden increase in tunnelling current. The tip was then retracted by 1.5 Å and translated in constant-height mode to the position corresponding to the desired conformation change. The retraction cut off the connection with the CN group, inducing *cis-trans* isomerization of the monomers within the molecular chain. Moreover, following the alignment at the alkyne–alkyne junction, the nanowire was disturbed via increased current and laterally manipulated to produce a bent conformation (Figure 5b), demonstrating excellent mechanical flexibility. Furthermore, Gross et al. realized intramolecular Glaser-type coupling via tip manipulation [102]. The precursor was deposited on a Cu(111) substrate covered with bilayer NaCl at 10 K, yielding three conformations, i.e., *trans*-1, (*P*)-*cis*-1, and (*M*)-*cis*-1 (Figure 5d–g). Owing to the constrained operational range of the tip, manipulation primarily targeted the *cis* isomer. By positioning the tip above the target molecule and applying a voltage pulse (>4.5 V) sustained for several seconds, the reaction was triggered, and product 2 was obtained successfully. In addition, Kim et al. employed voltage pulses at enyne linkages, formed by the addition reaction of terminal alkynes (as discussed in Figure 2d) [72], to induce in situ dehydrogenation, and eventually produced a graphdiyne-like chain.

## 6. Reactions Mediated by Extrinsic Components

In addition to the aforementioned intrinsic factors (including metal surfaces and molecular precursors) which are originally involved in the molecular systems, extrinsic components (which refer to substances introduced externally into the target reaction systems instead of inherent or reaction-generated constituents) have shown great promise in influencing on-surface reactions. The introduction of extrinsic components into on-surfaces reactions not only modulates reaction selectivity [56,58,59,103,104,105] and enhances stability but also enables multicomponent synergistic effects that optimize reaction kinetics [61]. Recent advances have demonstrated the efficacy of oxygen [89,105,106] and aromatic halides [39,40,76,93] in regulating terminal alkyne reactions, especially the latter with a radical transfer mechanism being elucidated to facilitate efficient terminal alkyne activation.

**Figure 6:**
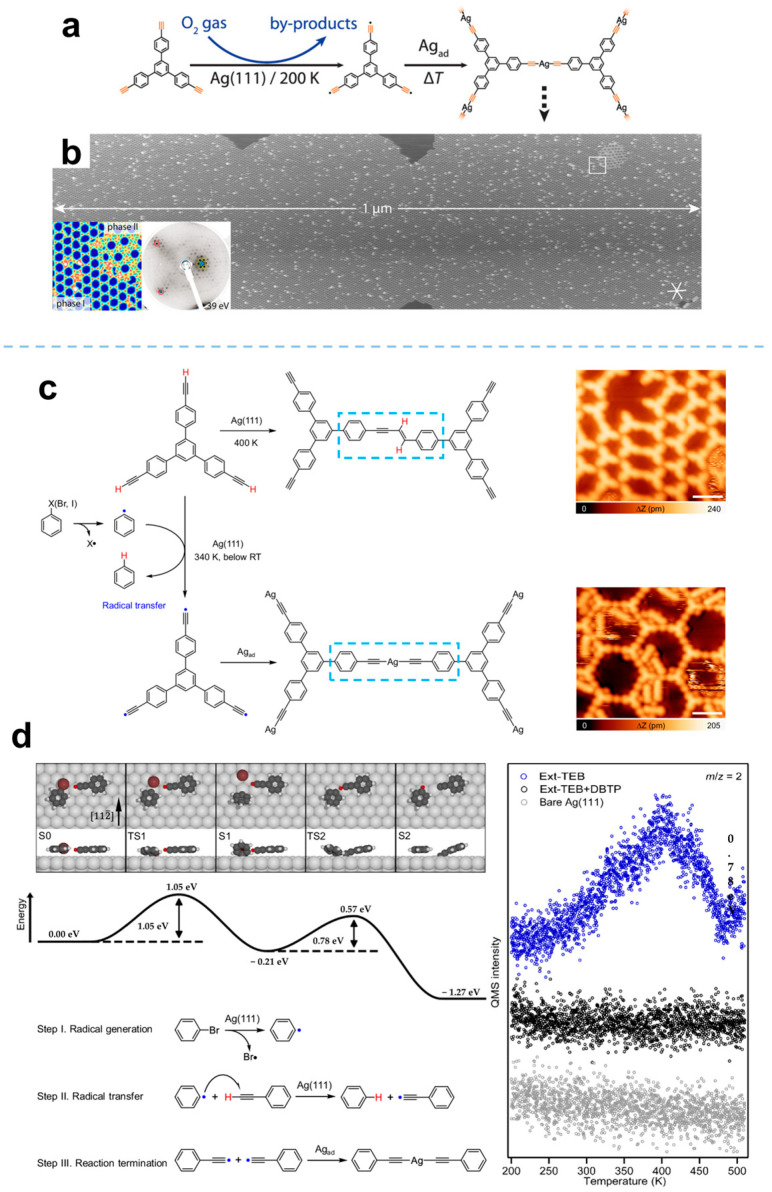
Reactions of terminal alkynes mediated by extrinsic components. (**a**) Schematic of reactions mediated by oxygen. (**b**) Large-scale STM image showing micro-scale organometallic network with LEED pattern. Copyright 2019 American Chemical Society [106]. (**c**) Schematic of the radical transfer reaction pathway. Scale bars are 2 nm. (**d**) DFT calculation of radical transfer reaction complemented with TPD spectra of H_2_ [40].

Oxygen, a highly reactive oxidizing gas species, has been reported to catalyze inert C–H activation by lowering the reaction barriers [56,58,105], thereby giving access to reaction products under milder conditions. This catalytic capability also motivates its introduction as an extrinsic component in the facilitation of terminal alkyne reactions. Zhang et al. pioneered the introduction of oxygen species to mediate terminal alkyne reactions on Ag(111) [106], which induced the C(*sp*)–H activation and enabled the highly selective construction of micrometer-scale two-dimensional organometallic networks (Figure 6a,b). Intact Ext-TEB molecules precovered Ag(111) sample was initially exposed to ~6000 L oxygen at 200 K, forming densely packed molecular islands. Sequential annealing to 250 K and 300 K triggered structural evolution from monomers to organometallic dimer, trimer, and hexamer structures. Further annealing at 375 K yielded a micrometer-scale two-dimensional organometallic network. Post-reaction XPS analysis confirmed the complete removal of oxygen species, which demonstrated the high effectivity and efficiency of the introduction of O_2_ into the reaction system. Applying this strategy, Barth’s group further incorporated Cu atoms into Ag-based organometallic networks, followed by annealing at 500 K to generate thermally stable Cu-based organometallic networks [107]. Moreover, through synergistic modulation of precursor design, substrate templating, and extrinsic O_2_, this approach enabled on-surface synthesis of enetriyne [89]. More interestingly, the combination of extrinsic components (i.e., O_2_, Na, and additional organic molecules) allowed the directional ring-chain interconversion of organometallic alkynyl–silver–alkynyl structures on Ag(111) [58], where the extrinsic interactions were revealed to be the key to such dynamic equilibrium.

Despite the established utility of terminal alkynes in constructing organometallic structures on Ag substrates under an oxygen atmosphere, the catalytic performance and reaction mechanism remain inadequately elucidated. Kim’s group filled this gap by intentionally introducing molecular oxygen and atomic oxygen onto Ag(111) and Au(111) [105], and elucidated both associative and dissociative C–H activation mechanisms for terminal alkynes. This work also revealed that while oxygen species were able to facilitate organometallic structure formation based on DEBP molecules on Au(111), the efficiency was substantially lower than that on Ag(111). Accordingly, oxygen-assisted C–H activation of alkynes has emerged as a prevalent strategy for constructing organometallic structures on Ag(111) substrates [58,107].

Apart from the O_2_-promoted alkyne reactions, other extrinsic species have also been demonstrated to be versatile in regulating reactions. As early as 2018, Wu et al. postulated that aromatic halides promoted the C–H activation of terminal alkynes [108], though the mechanistic details remained unresolved at that time. By further precisely controlling stoichiometric ratios of aromatic halides to terminal alkynes, Wu and his colleagues achieved highly selective alternating copolymerization [62]. They proposed a reaction pathway involving the dehalogenation of aromatic halides to form radicals and subsequent radical transfer to terminal alkynes. Concurrently, Chi et al. established a comprehensive radical transfer mechanism through systematic investigations and demonstrated a yield of almost 100% in this radical transfer process on Ag(111) [40]. The halogenated aryl molecules produced radicals via the dehalogenation reaction, and then alkynyl H quenched such aryl radicals along with the formation of terminal alkynyl radicals. Subsequently, radical-acquired terminal alkynes evolved to form regular alkynyl–metal–alkynyl structures with substrate adatoms. It is noteworthy that the dehalogenation reaction was the rate-determining step. Accordingly, C–Cl bonds exhibited such high activation barriers that radicals were difficult to be formed and terminal alkynes preferred to undergo the addition coupling reaction. In contrast, bromine and iodine substituents were much easier to detach, and terminal alkynes preferentially underwent radical transfer pathways to produce organometallic structures and passivated arenes. The corresponding experimental and computational process is briefly depicted in Figure 6c,d. Moreover, through the rational design of the steric hindrance of molecular precursors, Li et al. went a step further by synthesizing persistent yet highly reactive free radicals and achieved direct real-space observation of radical transfer mechanisms [39]. Application of this mechanism not only enhanced reaction selectivity but also enabled the synthesis of chiral polymers [92].

## 7. Conclusions and Outlooks

In conclusion, we summarize and categorize the assembly and reactions of surface-confined terminal alkynes from the perspective of mediated regulation strategies in this review. Supramolecular self-assembly, regulated by non-covalent interactions (e.g., C(*sp*)–H∙∙∙π, C(*sp*)–H∙∙∙N, C(*sp*)–H∙∙∙M), enables energetically favorable pre-assembly and molecular aggregation at low molecular coverages, significantly enhancing reaction efficiency. At the same time, the catalytic activity of substrates and their adsorption capacity for precursors critically influence reaction products. Metal substrates with different elements and miller indices have also been shown to yield distinct reaction products. In the absence of additional active functional groups or extrinsic components, pristine terminal alkynes generally undergo non-dehydrogenative addition reactions on Ag surfaces upon thermal treatments, while favoring [2+2+2] cyclization on Au surfaces. In contrast, Cu substrates, featured by their high reactivity, usually lead to the participation of free surface adatoms in the reaction procedures.

In the aspect of precursor design, incorporating functional groups (e.g., halogens and pyridines) into terminal alkynes usually provides additional non-covalent intermolecular interactions during self-assembly and extra reaction sites for subsequent reactions. This expands potential reaction pathways, suppresses specific side reactions, and enhances reaction selectivity. In addition, tip manipulation is another available regulation strategy which allows in situ modification and activation of structures. It not only enables monomeric conformational changes of homocoupled chains of terminal alkynes, but also realizes in situ C–H activation of terminal alkynes and dehydrogenation of coupled enyne connections to form graphdiyne-like chains. In the end, the introduction of extrinsic components (e.g., oxygen and aromatic halides) has shown effective reduction in the thermal activation temperatures. Consequently, complete dehydrogenation with high selectivity has usually been achieved, especially on Ag(111), where large-scale organometallic structures and alternating copolymer products can be obtained.

On the basis of the aforementioned efforts devoted to the reactions of surface-confined terminal alkynes, the following aspects should be further considered. (i) A more comprehensive understanding of these reaction regulation strategies should be critical for guiding experimental phenomena and achieving target products. (ii) Despite the existence of highly selective regulatory approaches, terminal alkynes usually exhibit numerous side reactions on surfaces, serving as a well-established challenge which should be overcome in the future to fulfill its potential applications in the construction of graphdiyne-related nanostructures. (iii) Furthermore, the synthesis of multifunctional carbon nanostructures with minimal defects and excellent properties (e.g., graphdiynes) based on terminal alkynes remains a significant challenge.

Therefore, such challenges necessitate: (i) Exploration of diverse reaction conditions. Apart from these well-established regulation strategies, other reaction conditions, such as varied atmospheres and temperature gradients, should be further explored in future work to assess their impact on the corresponding reactions of terminal alkynes. This exploration aims to uncover novel reaction phenomena and synthetic strategies, expanding possibilities for synthesizing advanced carbon nanostructures and nanomaterials. (ii) Integration of multiple synthetic strategies. Combining these strategies including supramolecular assembly, metal substrate regulation, precursor design, tip manipulation, and extrinsic component introduction may leverage their respective advantages to achieve more precise and complex synthesis, which requires systematic investigation to establish regulation rules. (iii) Characterization and modulation of the properties of nanostructures. With the increasing emergence of low-dimensional nanomaterials synthesized through on-surface reactions of terminal alkynes, deeper understanding and further modulation of their physicochemical properties, including but not limited to electronic, optical, and magnetic properties, are essential. Unraveling the relationships between structures and properties will establish a robust experimental and theoretical foundation for the coming application development.

## Data Availability

Not applicable.
